# Genomic characterization of large rearrangements of the *LDLR *gene in Czech patients with familial hypercholesterolemia

**DOI:** 10.1186/1471-2350-11-115

**Published:** 2010-07-27

**Authors:** Radan Goldmann, Lukáš Tichý, Tomáš Freiberger, Petra Zapletalová, Ondřej Letocha, Vladimír Soška, Jiří Fajkus, Lenka Fajkusová

**Affiliations:** 1 University Hospital Brno, Centre of Molecular Biology and Gene Therapy, Černopolní 9, CZ-62500 Brno, Czech Republic; 2 Centre for Cardiovascular Surgery and Transplantation, Pekařská 53, CZ-656 91 Brno, Czech Republic; 3 St. Anne's University Hospital Brno, Department of Clinical Biochemistry, Pekařská 53, CZ-656 91 Brno, Czech Republic; 4 Masaryk University, Faculty of Science, Institute of Experimental Biology, Department of Functional Genomics and Proteomics, Kamenice 5, CZ-62500 Brno, Czech Republic

## Abstract

**Background:**

Mutations in the *LDLR *gene are the most frequent cause of Familial hypercholesterolemia, an autosomal dominant disease characterised by elevated concentrations of LDL in blood plasma. In many populations, large genomic rearrangements account for approximately 10% of mutations in the *LDLR *gene.

**Methods:**

DNA diagnostics of large genomic rearrangements was based on Multiple Ligation dependent Probe Amplification (MLPA). Subsequent analyses of deletion and duplication breakpoints were performed using long-range PCR, PCR, and DNA sequencing.

**Results:**

In set of 1441 unrelated FH patients, large genomic rearrangements were found in 37 probands. Eight different types of rearrangements were detected, from them 6 types were novel, not described so far. In all rearrangements, we characterized their exact extent and breakpoint sequences.

**Conclusions:**

Sequence analysis of deletion and duplication breakpoints indicates that intrachromatid non-allelic homologous recombination (NAHR) between *Alu *elements is involved in 6 events, while a non-homologous end joining (NHEJ) is implicated in 2 rearrangements. Our study thus describes for the first time NHEJ as a mechanism involved in genomic rearrangements in the *LDLR *gene.

## Background

Familial hypercholesterolemia (FH) is an autosomal dominant disease, caused predominantly by variants in the low density lipoprotein receptor (*LDLR*) gene. Pathogenic alternations in the LDLR protein cause a lack of functional receptors for LDL particles on the liver cell surface and give rise to increased serum LDL-cholesterol levels. The high LDL-cholesterol level frequently gives rise to tendon xanthomas, xanthelasmas, arcus lipoides corneae, and accelerated atherosclerosis resulting from cholesterol deposition in the arterial wall, thereby increasing the risk of premature coronary heart disease. The frequency of heterozygous FH in most populations is about 1/500, homozygous FH is rare (≤ 1/1000,000) [1]. The identification and treatment of FH patients and their affected relatives with effective lipid-lowering agents is important and as this has been shown to significantly reduce both coronary morbidity and mortality [2,3]. Genetic testing is the preferred diagnostic method in FH families because it provides an unequivocal diagnosis [1,4,5]. The *LDLR *gene is localized at 19p13.2, is composed of 18 exons spanning 45 kb, the transcript is 5.3 kb long and encodes a peptide containing 860 amino acids [6]. *LDLR *mutations have been reported along the whole length of the gene in FH patients from around the world. At present, the number of identified unique *LDLR *allelic variants is over 1000: 65% of the variants are DNA substitutions, 24% small DNA rearrangements (< 100 bp) and 11% large DNA rearrangements (> 100 bp) http://www.ucl.ac.uk/ldlr/Current/index.php?select_db=LDLR and [7].

Genesis of large DNA rearrangements in the *LDLR *gene is frequently associated with *Alu *elements, which are highly abundant in this particular locus [6,8,9]. Publication of the human genome DNA sequence has revealed that there are 98 *Alu *repeats within the *LDL*R gene (95 in intronic sequences and 3 in the 3'untranslated region) and *Alu *repeats accounted for 65% of *LDLR *intronic sequences [10].

*Alu *is the most abundant short interspersed nuclear element (SINE) of the human genome, occupying 10% of the genome content with a copy number estimated to be at least 1.3 million [11]. Consensus *Alu *sequence is approximately 300 bp in length, and consists of two similar, but distinct monomers. The longer right *Alu *monomer contains a 31 bp insert absent from the left *Alu *monomer. A functional RNA polymerase III promoter is present in the left monomer, but is absent from the right monomer [12,13]. *Alu *sequences are regarded as retrotransposons that have inserted into the human genome via a single-stranded RNA intermediate generated by RNA pol III transcription [14]. The *Alu *dimer is usually followed by a 3'A-rich region, a typical feature of SINEs, and the two monomers are separated by a middle A-rich region, an obvious remnant of an ancestral monomeric *Alu*'s 3'A-rich tail [15].

Throughout *Alu *evolution, the source gene(s) accumulated mutations that were incorporated into the new copies made, creating new *Alu *subfamilies. Therefore, the *Alu *family is composed of a number of distinct subfamilies characterized by a hierarchical series of mutations that result in a series of subfamilies of different ages [16-20].

*Alu *repeat dispersion throughout the genome offers many opportunities for homologous recombinations. Nonallelic homologous recombination (NAHR) is the most common mechanism underlying disease associated genome rearrangements. NAHR can use either region-specific low-copy repeats or repetitive sequences (e.g., *Alu*) as homologous recombination substrates [21,22]. Another recombination mechanism causing genomic disorders is nonhomologous end joining (NHEJ). This process involves the double strand breakage of DNA followed by end joining in the absence of extensive sequence homology [23-25]. NHEJ is associated with very short stretches of sequence identity (a few bp) between the two ends of the breakpoint junctions [22,26,27].

In this study, we present results of analyses of large genomic rearrangements in Czech FH patients detected using Multiple Ligation dependent Probe Amplification (MLPA). In set of 1441 unrelated FH patients, large genomic rearrangements were detected in 37 probands. We found 8 different types of rearrangements, from them 6 types were novel, not described so far. In all rearrangements, we characterized their exact extent and breakpoint sequences. The results showed that 6 events were products of NAHR between *Alu *repeat sequences. The remaining 2 events apparently originated from NHEJ.

## Methods

### Patients

One thousand nine hundred and forty five probands with probable or definite diagnosis of FH, submitted to the database of the MedPed (Make Early Diagnoses to Prevent Early Deaths) project in the Czech Republic, were included into the study. MedPed is an international project joining together experts from more than 30 countries of the world. In the Czech Republic, the project is coordinated by the Czech Society for Atherosclerosis. Experimental research reported in this study has been performed with the approval of the Ethical Committee of the General University Hospital in Prague, the Czech Republic, and is in compliance with the Helsinki Declaration. All patients gave their informed consent with their participation in the study, which is a part of each patient's personal documentation. The text of the informed consent is available at: http://www.athero.cz/user_data/zpravodajstvi/obrazky/File/medped/informovany_souhlas.pdf The patient file in our study include a) patients with untreated total and/or LDL cholesterol serum levels above the 95^th ^percentile of age, sex and population specific values; b) patients with elevated total and LDL cholesterol in serum but untreated levels unavailable or not exceeding the 95^th ^percentile of age, sex and population specific values, and, in addition, with high clinical suspicion of FH based on personal history and/or family history of premature coronary heart disease and/or elevated total and LDL cholesterol serum levels in the first degree relatives.

DNA analysis of FH patients is divided into several consecutive steps: 1) PCR-RFLP detection of the most common mutation in the *APOB *gene (p.Arg3527Gln) [28,29]; 2) PCR-RFLP detection of the most common mutations in the *LDLR *gene (p.Gly592Glu, p.Asp266Glu, and p.Arg416Trp); 3) PCR-sequencing of *LDLR *exon 4 (the exon with the greatest occurrence of mutations in Czech FH patients); 4) MLPA analysis of all *LDLR *exons; 5) PCR-sequencing of the promoter and *LDLR *exons 1, 5, 6, 9, 10, 12, 14; and 6) PCR-denaturing high performance liquid chromatography of *LDLR *exons 2, 3, 7, 8, 11, 13, 15, 16, 17, and 18, followed by sequencing of positively tested regions.

The break of DNA analysis in case of a mutation finding depends on personal and family history of hypercholesterolemia, the presence of tendon xanthomas, xanthelasmas, early coronary artery disease and premature coronary heart disease. The DNA analysis continues in cases when i) a phenotype manifestation could be associated with the presence of two *LDLR *mutations or ii) a detected missense mutation is new with hardly predicted effect on the protein structure and function. This diagnostic process is common in FH diagnostics [30,31]. Only data obtained by MLPA analysis are present in this study.

### Analysis of deletion and duplication breakpoints in the *LDLR *gene

DNA was isolated according to the standard salting-out method. MLPA was performed using SALSA MLPA KIT P062-C1 LDLR (MRC-Holland), according to the manufacturer's instruction, and analysed on CEQ 8000 Genetic Analysis System (Beckman Coulter). To characterize the precise locations of genomic breakpoints, a number of amplifications and PCR product analyses were performed. Primers for initial long-range amplifications are given in Table 1 together with approximate sizes of PCR fragments of mutated alleles and nested primers for precise determination breakpoints using DNA sequencing. Long-range PCR were performed using Expand Long Template PCR System Kit (Roche) and PCR amplifying fragments around breakpoints using AmpliTaq Gold polymerase (Applied Biosystems). PCR products were purified and sequenced on ABI PRISM 310 DNA-sequencer (Applied Biosystems). Repetitive sequences were identified using RepeatMasker version-3.1.5 available at http://www.repeatmasker.org/cgi-bin/WEBRepeatMasker.

**Table 1:** Primers for *LDLR *breakpoint analysis

*Mutation at cDNA level*	*Primers for long-range PCR* *(5'→ 3'direction)*	Size* (kb)	*Primers for precise breakpoint determination* *(5'→ 3'direction)*
promoter_ex2del	F: TGTCGCAAATGGCATAAGGAA R: CGGATTTGCAGGTGACAGACA	2.0	F: AAGGCTGCAGTGAAGTATGATGG R: GAGACGGAGTCTCACTCTGTCG
exon2_6dup	F: AGTTCAAGTGTCACAGCGGC R: GTCTTGGCACTGGAACTCGT	8.0	F: AGTTCAAGTGTCACAGCGGC R: CAAGGTTGGCGTTTTTCATATT
exon3_12del	F: CCAGAAGATTCCAGAAATTTCCAG R: CCTTTCTCCTTTTCCTCTCTCTCA	3.5	F: TGGCTCACTGCAAGCTCCG R: AGGCTGGAGTCCAGTGGTACC
exon4_8dup	F: CAAGTGCCAGTGTGAGGAAGG R: CCCTTGGAACACGTAAAGACCC	2.5	F: CACGTGACTTCAAGGGGTTAAAG R: TTCTCTAAAATGCTTGGGACCA
exon5_10del	F: CACCTGCATCCCCCAGCTGTGGGC R:TGGCTGGGACGGCTGTCCTGCGAAC	3.0	F: TTTGTACAGACACAGGCTGGTC R: CAGATGTCACCTGACAGGTACAG
exon9_14del	F: GGAGTGACTTCAAGGGGTTAAAG R: AGGTGGCTCAGGCTGGGC	0.5	F: GGAGTGACTTCAAGGGGTTAAAG R: AGGTGGCTCAGGCTGGGC
exon9_15del	F: CACGTGATCGTCCCGCCTA R: AAATTCTTGTCAACCTACTTGTGC	0.8	F: AAATTCTTGTCAACCTACTTGTGC R: CACGTGATCGTCCCGCCTA
exon16_18dup	F: CGTGAACATCTGCCTGGAGTC R: TCTTCTCATTTCCTCTGCCAGC	3.0	F: TCGTGTGTGTTGGGATGGGA R: ACCCCAGCCCCCAAACTAAA

F - forward primer; R - reverse primer; * size of PCR fragment of mutated allele. The genomic sequence of the *LDLR *gene was obtained from http://www.ucl.ac.uk/ldlr/LOVDv.1.1.0/refseq/LDLR_codingDNA.html.

## Results

For DNA analysis, 1945 FH probands were selected: 252 probands (13,0%) had the *APOB *mutation; 186 probands (9,6%) had the mutation p.Gly592Glu or p.Asp266Glu or p.Arg416Trp; 66 probands (3,4%) had a mutation in exon 4. 1441 patients were analyzed by MLPA and in 37 probands (1,9%) a deletion/duplication was detected. At present time, the DNA analysis continues in 1404 patients.

**Figure 1:**
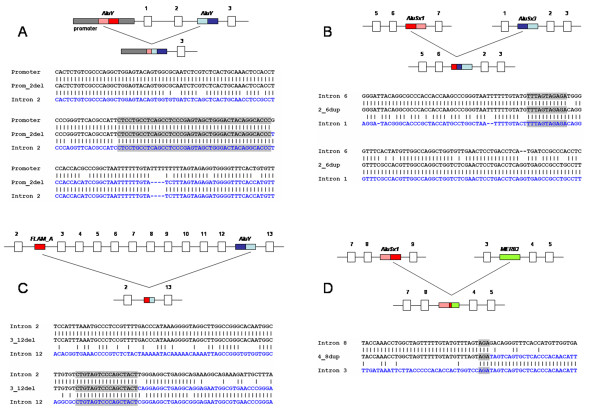
**Schematic illustration of rearrangements in the *LDLR *gene including DNA sequence of breakpoints**. **A**: promoter_exon2del, **B**: exon2_6dup, **C**: exon3_12del and **D**: exon4_8dup. Consensus *Alu *sequences are depicted as red and blue boxes, their monomer subunits are given in dark and light tones. Sense orientation is marked by a darker tone of the first monomer of the *Alu *consensus sequence, the opposite order marks antisense orientation. MER83 repeat is depicted as a green box. Grey boxes represent sequence overlaps between 5'end and 3' end of the reference sequence.

**Figure 2:**
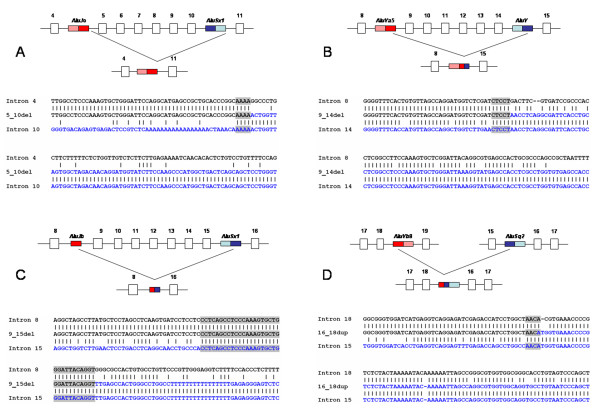
**Schematic illustration of rearrangements in the *LDLR *gene including DNA sequence of breakpoints**. **A**: exon5_10del, **B**: exon9_14del, **C**: exon9_15del and **D**: exon16_18dup. Consensus *Alu *sequences are depicted as red and blue boxes, their monomer subunits are given in dark and light tones. Sense orientation is marked by a darker tone of the first monomer of the *Alu *consensus sequence, the opposite order marks antisense orientation. Grey boxes represent sequence overlaps between 5' end and 3' end of the reference sequence.

Using MLPA, we found 8 types of large genomic rearrangements - 5 deletions and 3 duplications (Table 2). Six types of rearrangements were novel, so far not described: exon2_6dup, exon3_12del, exon4_8dup, exon5_10del, exon9_15del, and exon16_18dup (The terminology used should be read e.g., in exon2_6dup as duplication of exon 2 to exon 6). Using long-range PCR, PCR, and DNA sequencing, we analysed breakpoints of deletions and duplications identified in our FH patients. In Table 2, we show correct sizes of deletions and duplications together with terms of repetitive elements surrounding breakpoints. Schematic illustration of recombination events are given in Figure 1 and 2. As new rearrangements, we denote deletions/duplications which have not been described in literature so far in terms of exons involved. In this denotation, we do not take into account the exact sequence position of breakpoints determined in this work.

**Table 2:** Genomic characteristics of deletion and duplication breakpoints in the *LDLR *gene in Czech FH patients

Mutation at cDNA level	Mutation at DNA level	Deletion/duplication size	Recombination mechanism	Repetitive element 5'/class/family	Repetitive element 3'/class/family	No. of probands
promoter_2exondel	c. -1823_190+566del	13186bp	NAHR	AluY/SINE/Alu	AluY/SINE/Alu	1
**exon2_6dup**	**c**. **67+3968_940+296dup**	14228bp	NAHR	AluSx1/SINE/Alu	AluSx3/SINE/Alu	9
**exon3_12del**	**c.190+984_1846-1160del**	17604bp	NAHR	FLAM_A/SINE/Alu	AluY/SINE/Alu	1
**exon4_8dup**	**c.314-446_1187-386dup**	8119bp	NHEJ	AluSx1/SINE/Alu	MER83/LTR/ERV1	1
**exon5_10del**	**c. 695-67_1586+371del**	7636bp	NHEJ	AluJo/SINE/Alu	AluSx1/SINE/Alu	4
exon9_14del	c.1186+700_2141-545del	10291bp	NAHR	AluYa5/SINE/Alu	AluY/SINE/Alu	10
**exon9_15del)**	**c.1187-169_2312-790del**	14110bp	NAHR	AluJb/SINE/Alu	AluSx1/SINE/Alu	8
**exon16_18dup)**	c.2311+1941_*1216dup	7248bp	NAHR	AluYb8/SINE/Alu	AluSq2/SINE/Alu	3

Newly described rearrangements are in bold letters; NAHR: nonallelic homologous recombination; NHEJ: nonhomologous end joining.

NAHR was detected in six DNA rearrangements (promoter_exon2del, exon2_6dup, exon3_12del, exon9_14del, and exon9_15del, exon16_18dup). In four NAHRs (promoter_exon2del, exon2_6dup, exon9_14del, and exon16_18dup), extensive sequence identity was detected between the breakpoints. In all four cases, the rearrangements were caused by recombination between consensus *Alu *repeats and novel complete recombinant *Alu *sequence was formed in the mutation breakpoint. In contrast, sequence identity around breakpoints of rearrangements exon3_12del and exon9_15del was not so extensive like in previous cases. These mutations were caused by recombination between an *Alu *repeat in monomer status and a consensus *Alu *repeat (dimer status). The recombination between *FLAM_A *(free left *Alu *monomer, size: 133 bp) and *AluY *(size: 315 bp) was detected in exon3_12del. The recombination between *AluJb *(size: 137 bp) and *AluSx1 *(size: 311 bp) was identified in exon9_15del. The deletion breakpoints of both consensus *Alu *repeats were localised in right monomer and so novel complete monomer recombinant *Alu *sequence was formed in the mutation breakpoint. Promoter_exon2del, exon2_6dup, exon9_14del and exon9_15del were formed between *Alu *repeats in the antisense orientation, exon3_12del and exon16_18dup between *Alu *repeats in the sense orientation.

NHEJ was detected in two DNA rearrangements (exon5_10del and exon4_8dup). In exon5_10del, the breakpoint localized in intron 4 was present at the end of the *AluJo *repeat in antisense orientation, and the breakpoint localized in intron 10 was at the end of the *AluSx1 *repeat in the sense orientation. In exon4_8dup, the breakpoint in intron 3 was localized in the *MER83 *repeat (*ERV1 *family repeat) and the breakpoint in intron 8 in the *AluSx1 *sequence. There is no sequence homology between these repeats.

## Discussion

The 117 large DNA rearrangements are listed on http://www.ucl.ac.uk/ldlr/Current/index.php?select_db=LDLR[7]: 100 deletions and 17 duplications. In the view of 98 *Alu *repeats within the *LDLR *gene [10], it is probable that DNA rearrangement breakpoints are located inside of *Alu *repetitive sequences. In the set of our FH patients, we detected 37 large DNA rearrangements in the *LDLR *gene and performed the precise characterization of breakpoints in all types of deletions and duplications. Results define most of breakpoints inside of *Alu *repeats (except one localised in *ERV1 *repeat) and NAHR and NHEJ as responsible for these rearrangements. Our results thus demonstrate that *Alu *mediated recombination leads to massive disturbances in the structural and functional integrity of the *LDLR *gene region.

Promoter_exon2del is 13186 bp long and was detected in one Czech FH proband. Approximately 20 kb and 18 kb deletions of promoter, exon 1 and 2 were described previously [32,33]. Exon3_12del detected in one Czech FH proband was not described previously but deletions involving exon 3 were identified (exon3del [34], exon3_5del [35], exon3_6del [34], exon3_8del [36], and exon3_10del [37]). Exon5_10del was found in 4 Czech FH probands and was not described previously. Deletions encompassing exon 5 (exon5del and exon5_6del) were detected in studies [34,38], respectively. Exon9_14del was detected in 10 Czech FH probands. Niessen et al. found exon9_14del in Danish FH patients and performed also analysis of breakpoints. The correct size of the deletion described by Niessen was 9713 bp and both deletion breakpoints were localised in repetitive elements *AluSq *[39]. Exon9_14del determined in our FH probands sized 10291 bp and breakpoints were localised in repetitive elements *AluYa5 *and *AluY*. Exon9_10del and exon9_12del were also detected in literature [40]. Exon9_15del was present in 8 Czech FH probands and was not described previously.

All duplications detected in our FH patients were new, not described so far. Exon2_6dup was detected in 9 Czech FH probands, exon4_8dup was found in one Czech FH proband, and exon16_18dup was determined in 3 Czech FH probands. The duplications exon2_8dup, exon4_5dup, and exon16_17dup were described [41-43].

It is interesting that exon2_6dup and exon9_15del were not described in literature and on http://www.ucl.ac.uk/ldlr/Current/index.php?select_db=LDLR, but in Czech FH patients these are relatively frequent (9 and 8 probands, respectively).

In the above mentioned work, Nissen et al. [39] described 5 genomic deletions in the *LDLR *gene and defined the breakpoints of each deletion. The five deletions were flanked by *Alu *elements, supporting a mutation mechanism involving unequal homologous recombination between highly similar *Alu *elements. The deletion exon13_15del described by Nissen et al. was flanked by two *AluSg *elements and 15 bp had been inserted at the site of the deleted DNA. This short insertion did not show similarity to any interspersed repeats or any other DNA sequence in the LDLR gene. However, the sequence shows partial homology to several sites in human genome. It is possible to speculate, that in this particular case, the final sequence arrangement has been generated by a more complex mechanism of double strand break repair, involving several recombination steps (e.g., resection and invasion of one DNA strand to a site of a partial homology and its elongation, which was not followed by the single-strand annealing step of homologous recombination, but instead by synthesis-dependent NHEJ). Alternatively, this kind of deletion could have been produced by NHEJ alone, without previous steps of homologous recombination). However, this cannot be clearly distinguished from the final sequence.

In this respect it should be mentioned that deletion and duplication spectra as the outcomes of recombination events in a given genomic locus are influenced not only by the DNA sequence context in the region itself (e.g., abundance and orientation of repeats and their variability) [44,45], but also by epigenetic factors. It corresponds to the fact that it is chromatin template, not a naked DNA, which is a subject of recombination. In the particular case of *Alu *repeats, the role of heterochromatic marks such as DNA methylation, or histone H3K9 methylation in suppression of recombination by these elements has been suggested in recent studies [46-48].

## Conclusions

Eight different types of large genomic rearrangements were detected in the *LDLR *gene, from them 6 types were novel, not described so far. Sequence analysis of deletion and duplication breakpoints indicates that both intrachromatid non-allelic homologous recombination (NAHR), and non-homologous end joining (NHEJ) are involved in *LDLR *genomic rearrangements. While NAHR has been described in relation to the *LDLR *gene, this study as the first describes NHEJ in *LDLR *genomic rearrangements.

## Competing interests

The authors declare that they have no competing interests.

## Authors' contributions

RG performed detection of deletions/duplications in the *LDLR *gene using MLPA and detailed characterization of breakpoints, LT performed detection of deletions/duplications in the *LDLR *gene using MLPA and administrated database of patients, PZ and OL performed molecular analysis of the *LDLR *gene using PCR-RFLP, DNA sequencing and denaturing high performance liquid chromatography. TF and VS performed clinical examination, selection of patients with suspicion for familial hypercholesterolemia, and blood collections for DNA isolation. LF designed and coordinated the study and has been involved in evaluation of results and manuscript preparation. JF has been involved in evaluation of results and manuscript preparation. All authors read and approved the final manuscript.

## Pre-publication history

The pre-publication history for this paper can be accessed here:

http://www.biomedcentral.com/1471-2350/11/115/prepub
